# Effects of a Whole-School Health Intervention on Clustered Adolescent Health Risks: Latent Transition Analysis of Data from the INCLUSIVE Trial

**DOI:** 10.1007/s11121-021-01237-4

**Published:** 2021-04-21

**Authors:** G. J. Melendez-Torres, Elizabeth Allen, Russell Viner, Chris Bonell

**Affiliations:** 1 Peninsula Technology Assessment Group, College of Medicine and Health, University of Exeter, Exeter, UK; 2 Department of Medical Statistics, London School of Hygiene and Tropical Medicine, London, UK; 3 Department of Social Science, UCL Institute of Child Health, London, UK; 4 Department of Public Health, Environments and Society, London School of Hygiene and Tropical Medicine, London, UK

**Keywords:** Restorative practice, Latent transition analysis, Randomized trial, Bullying

## Abstract

Whole-school interventions are a promising approach to preventing bullying and aggression while promoting broader health. The main analyses from a trial of the INCLUSIVE whole-school intervention reported reductions in bullying victimisation but not aggression and improved mental well-being. Latent transition analysis can examine how interventions ‘move’ people between classes defined by multiple outcomes over time. We examined at baseline what classes best defined individuals’ bullying, aggression and mental well-being and what effects did the intervention have on movement between classes over time? INCLUSIVE was a two-arm cluster-randomised trial with 20 high schools per arm, with 24-month and 36-month follow-ups. We estimated sequential latent class solutions on baseline data. We then estimated a latent transition model including baseline, 24-month and 36-month follow-up measurements. Our sample comprised 8179 students (4082 control, 4097 intervention arms). At baseline, classes were (1) bullying victims, (2) aggression perpetrators, (3) extreme perpetrators and (4) neither victims nor perpetrators. Control students who were extreme perpetrators were equally likely to stay in this class (27.0% probability) or move to aggression perpetrators (25.0% probability) at 24 months. In the intervention group, fewer extreme perpetrators students remained (5.4%), with more moving to aggression perpetrators (65.1%). More control than intervention extreme perpetrators moved to neither victims nor perpetrators (35.2% vs 17.8%). Between 24 and 36 months, more intervention students moved from aggression perpetrators to neither victims nor perpetrators than controls (30.1% vs 22.3%). Our findings suggest that the intervention had important effects in transitioning students to lower-risk classes.

## Introduction

Adolescence is a time typically characterised by good physical health but the emergence of risk behaviours, including bullying and aggression, as well as common mental health problems, with important consequences for individuals’ current as well as adult mental and physical health. Such risks and problems tend to cluster within individuals (Meader et al., 2016), with clustering of multiple risks being associated with particularly poor health in adulthood (Akasaki et al., 2020). Whole-school interventions are a promising approach to preventing bullying and aggression while also promoting broader health (Smith et al., 2004; Vreeman & Carroll, 2007). These aim to render school environments more health promoting and supportive of individual social and emotional development in order to achieve benefits across health outcomes (Bonell et al., 2019). Restorative practice offers another means to prevent bullying and aggression by preventing or resolving school-based conflicts between students or between staff and students (Morrison, 2005). Victims communicate to perpetrators the harms done, and perpetrators acknowledge and amend their behaviour. Primary restorative practice aims to prevent incidents while secondary restorative practice aims to resolve incidents. Restorative practice is increasingly used within schools, with promising results from non-experimental evaluations (Kane et al., 2007)

Informed by previous evidence and theory (Flay et al., 2004; Markham & Aveyard, 2003; Patton et al., 2006), INCLUSIVE was a whole-school health intervention that included training for staff on using restorative practice to address school conflict, student participation in decision-making via a school-level student/staff action group and a social and emotional learning classroom curriculum as key components. This was hypothesised to reduce bullying victimisation and perpetration of aggression (twin primary outcomes) and improve various secondary health outcomes including mental well-being. The main outcome evaluation reported evidence of effectiveness on bullying victimisation but not perpetration of aggression as well as effects on various secondary outcomes including mental well-being. Effects were apparent at final 36-month follow-up but not at interim 24-month follow-up (Bonell et al., 2018). These analyses did not examine the effects of the intervention on how individuals experienced multiple risks at these different timepoints.

Intervention effectiveness is generally assessed in terms of differences between intervention and control groups in discrete endpoints at a particular timepoint or in change trajectories. These can be described as variable-centred approaches, in that they focus on changes in the values of individual variables. However, effectiveness can also be examined in terms of transitions between classes (Williams et al., 2015). Latent transition analysis (LTA), which is an extension of latent class analysis or latent profile analysis, can be regarded as a person-centred alternative in which people are described and classified in terms of their values on several variables. This enables examination of how interventions ‘move’ people between classes defined by one or more outcomes. LTA is a promising approach for exploring the effects of interventions, such as INCLUSIVE, which primary trial analyses indicate as effective on a range of outcomes with effects emerging over time. While variable-centred approaches capture mean differences in outcomes, supporting direct inference on expected changes in population means, person-centred approaches can illuminate who benefits from interventions, and how, with respect to multiple outcomes in the same analysis.

Previous LTAs of interventions to prevent adolescent bullying as well as dating violence have found that interventions were effective in moving people from classes defined by involvement in bullying perpetration, victimisation or joint perpetration-victimisation into classes defined as no or low perpetration or victimisation (Jenson et al., 2013; Williams et al., 2015). LTAs not used to evaluate interventions have also found joint victimisation-perpetration, victimisation only or neither in adolescents (Davis et al., 2020), or that joint victimisation-perpetration could be understood to be primarily relational or overall (Ettekal & Ladd, 2019). We undertook this LTA to add to our understanding of the effects of the INCLUSIVE intervention in terms of moving individuals between groups over 24- and 36-month follow-up. Unlike previous such analyses undertaken in trial contexts (Jenson et al., 2013; Williams et al., 2015), we included in our latent transition model measures examining different risk behaviours (aggression as well as bullying) as well as mental well-being in order to assess intervention effects on the individual clustering of multiple risks and problems. Our research questions were as follows: at baseline, what classes best defined individuals’ reported bullying victimisation, perpetration of aggression and mental well-being and what effects did the intervention have on individuals’ movement between classes over time?

## Methods

Trial methods for INCLUSIVE have been published elsewhere (Bonell et al., 2018). In summary, this was a two-arm cluster-randomised trial with 20 state secondary schools in each arm, with baseline, 24-month and 36-month follow-ups. Schools were broadly representative of schools in south-east England but excluded private schools, schools for those with learning disabilities or schools judged unsatisfactory by the national school inspectorate. The trial took place between 2014 and 2017, and students were surveyed at the end of year 7 (age 11–12) at baseline and at 24- and 36-month follow-up at the ends of years 9 (age 13–14) and 10 (age 14–15). The trial was approved by the University College London ethics committee (ref. 5248/001). Written, informed consent was sought from head teachers for allocation and intervention, and from individual students for survey participation. Parents were informed of the study and could withdraw their children from research if they wished. Student completed surveys in classrooms under examination conditions facilitated by trained researchers with teachers present but unable to read student responses.

Schools randomly allocated to the intervention group were provided with several components. All staff received introductory 2-h training on addressing school conflict, focusing on restorative practice in classrooms to prevent and respond to minor incidents; selected staff received 3-day training on implementing restorative conferences to address more serious incidents. Schools were also provided with an intervention manual and lesson plans and presentation slides for social and emotional learning lessons, informed by the Gatehouse Project curriculum (Patton et al., 2003). Schools received annual reports on student needs (drawing on questionnaire surveys with students) in relation to bullying, aggression and school experiences and comparing these to other schools receiving the intervention. Finally, an external facilitator with experience of school leadership guided action groups. Schools used these to implement restorative practice within classrooms and conferences to prevent and resolve violence and other conflict within the school; action groups comprising around six students and six staff, and meeting six times per year to review student needs data to decide local priorities and coordinate intervention activities; and 5–10 h per year of classroom education for students in years 8–10 (age 12–15) on social and emotional learning delivered by teachers. Control schools continued with usual practices concerning discipline, student participation and classroom curricula. The intervention ran for the full 3 years of the trial period, with a focus on embedding key functions into school processes.

In line with UK Medical Research Council guidance on evaluating complex interventions (Moore et al., 2015), we conducted a process evaluation assessing fidelity in all schools. In the first 2 years of intervention, we scored fidelity out of eight points for each school, evaluating whether five or more staff received in-depth training; action groups met six times per year; policies and rules were reviewed; locally decided actions were delivered; members assessed that action groups had a good or very good range of members; members assessed that action groups were well or very well led; schools provided at least 5 h or two modules of our curriculum per year; and at least 85% of staff reported that staff responded to conflict by talking to those involved to help them get on better. In the third year, we scored fidelity using a narrower range of data because the research teams had less access to schools. Schools were assessed out of four on whether action groups met six times; local decisions were implemented; schools delivered at least 5 h or two modules of the curriculum; and at least 85% of staff reported that they responded to trouble by talking to those involved.

Fidelity to the intervention varied over time and between schools. The median fidelity score for the first and second years was 6/8 with an inter-quartile range (IQR) of 5–7, and the median was 1/4 (IQR 0–3) in the third year. In the third year, 15 schools continued with restorative practice. Interviews and focus groups with staff suggested that schools commonly incorporated what they regarded as the most useful action group elements into existing school systems in this final year. Higher fidelity of implementation in the first 2 years but not the third year was associated with lower bullying victimisation at 24 months (Bonell et al., 2018).

The outcomes considered in this analysis are measures of bullying victimisation, perpetration of aggression and mental well-being. For bullying victimisation, we used the Gatehouse Bullying Scale, a 12-item self-reported measure of bullying frequency, and the upset caused by this bullying (Bond et al., 2007). Bullying behaviours included in this measure were teasing, name-calling, rumours, being left out, physical threats or actual violence (for example, *I have been deliberately left out*), in the last 3 months. Items are summed to create a scale score between 0 and 12, with higher values indicating higher levels of victimisation. At baseline, the scale had Cronbach’s alpha of 0.73, at 24 months, 0.72 and at 36 months, 0.71. For perpetration of aggression, we used the Edinburgh Study of Youth Transitions and Crime school misbehaviour subscale, which measures frequency and type of aggression towards peers or teachers (Smith, 2006), for example, *I hit or kicked another student*, *I threatened a teacher*, *I damaged school property*, each with a Likert scale from 0 (hardly ever/never) to 3 (most days). Items are summed to create a scale from 0 to 39, with higher values indicating higher levels of aggression. At baseline, the scale had Cronbach’s alpha of 0.87, which was consistent over follow-up waves (0.88 at 36 months). Finally, for mental well-being, we used the Short Warwick-Edinburgh Mental Well-being Scale, which has been validated for use in this age group (Clarke et al., 2011). This scale includes both functional and psychological aspects of mental well-being (as opposed to specific items relating to psychopathology), and ranges from 7 to 35, with higher values reflecting better mental well-being. Example items include *I’ve been feeling optimistic about the future* and *I’ve been feeling close to other people*, each with a Likert scale from 1 to 5 reflecting frequency (from ‘none of the time’ to ‘all of the time’). At baseline, the scale had Cronbach’s alpha of 0.83, improving to 0.89 at 36 months.

Our analysis procedure unfolded in several steps. All analyses accounted for clustering by school. First, we estimated sequential latent class solutions on baseline data, using all three outcome measures (victimisation, aggression and mental well-being) with normal link functions and setting variances for the same outcome measure equal between classes. This mean that latent classes (or latent profiles, given that manifest indicators were continuous) were interpreted on the basis of the conditional means of indicators in each class. We chose the optimal latent class solution on the basis of interpretability (did conditional mean estimates in each class generate an interpretable class solution?), balanced with the Bayesian information criterion (BIC) frontier, the scaled relative entropy frontier, and the Vuong-Lo-Mendell-Rubin adjusted likelihood ratio test (or VLMR-LRT) (Lo et al., 2001). The BIC frontier captures how improvements in model fit trail off with increasing model complexity, while the scaled relative entropy is analogous to an *R*^2^ from a multiple regression and captures how accurately and precisely latent class models classify individuals. The VLMR-LRT estimates whether a latent class solution with *k* classes offers an improvement in fit over a latent class solution with *k − *1 classes. We did not use the bootstrap likelihood ratio test as this is not available for clustered data.

After identifying an optimal number of classes based on baseline data, we estimated models with the same number of classes at both follow-up waves and inspected results for metric similarity. While we regarded that due to sample size, visual inspection for metric invariance was more meaningful, we undertook a metric invariance test using a multiparameter Wald test for equality of indicator means for each class between measurement waves in a latent transition model with no covariates. A non-significant metric invariance test suggests that classes are not significantly different with respect to indicator means across each follow-up time; however, given the large sample size, even small deviations may yield a significant result.

We then estimated another latent transition model including baseline, 24-month and 36-month follow-up measurements in the same model, and constraining classes to have equal scores and equal variances on each outcome measure over all waves (i.e. assuming metric invariance). This was required to avoid singularity of the information matrix. We did not assume invariance in transition probabilities between both sets of waves given that the intervention may have generated different transition probabilities over time and given the unequal time spacing between waves. We used intervention as a baseline covariate to account for any baseline differences, and estimated how intervention allocation related to the probability of transition from one class to the next. Conceptually, this means that intervention status was included in three different structural regression equations: the first is a multinomial logistic regression predicting class assignment at baseline on the basis of intervention status; the second is a multinomial logistic regression predicting class assignment at 24 months on the basis of class assignment at baseline, intervention status and the interaction of class assignment at baseline with intervention status; and a multinomial logistic regression predicting class assignment at 36 months on the basis of class assignment at 24 months, intervention status and the interaction of class assignment at 24 months with intervention status. We used a multiparameter Wald test to estimate whether intervention was a meaningful predictor of transitions between classes. In this test, we estimated jointly whether coefficients in the second and third regression equations that related to intervention status—that is, ‘main effects’ of intervention status in predicting class assignment and interaction effects of intervention status with class assignment—were equal to 0. A significant test thus suggests that intervention caused differences in transition probabilities between classes. For both intervention and control arms, we extracted transition matrices describing the probability of students moving classes at the next follow-up wave, conditional on current class membership. This resulted in four matrices: baseline to 24 months for intervention and for control and 24 to 36 months for intervention and for control.

Students who were missing for one or two waves of measurement were included using full information maximum likelihood. This was an appropriate choice as it meant all available data were included, while acknowledging that some students would enter and leave schools over the measurement period. Models were estimated using Mplus version 8 (Muthén & Muthén, 2017).

## Results

Of 7121 registered students, 6667 (93.6%) provided data at baseline: 3320 (94.4%) of 3516 in the intervention group and 3347 (92.8%) of 3605 in the control group. All schools participated in the follow-up surveys at 24 months and 36 months; the numbers of students who completed the questionnaires at baseline, 24 months and 36 months were similar in each group. Student and school characteristics and outcomes at baseline were well balanced across arms. The analysis sample for this study comprised 8179 students, of whom 4082 were in control schools and 4097 were in intervention arms. At baseline, the average level of bullying victimisation was 1.97, with variance 6.05; the average level of aggression was 2.92, with variance 23.12; and the average level of mental well-being was 24.22, with variance 34.92.

**Fig. 1:**
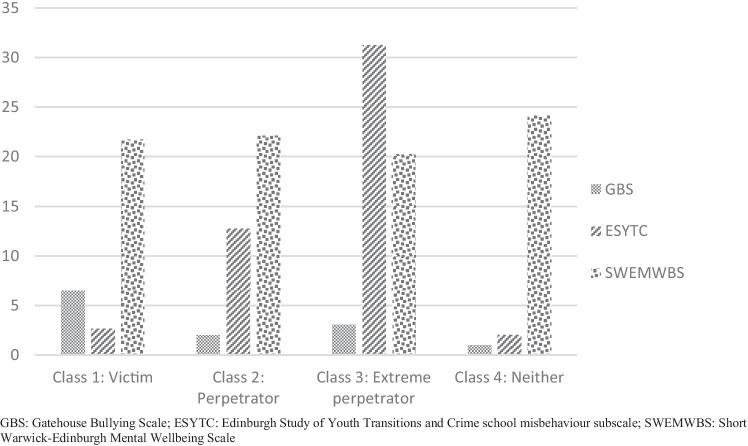
Overall latent classes

We tested between 1 and 6 latent class solutions (Table 1). Models with more than six classes failed to converge, even with 100 starting values and 40 followed up to optimisation. Improvements in BIC tapered after four classes, as did changes in scaled relative entropy. A six-class solution was worse than a five-class solution on BIC and VLMR-LRT. While the BIC preferred a five-class solution, scaled relative entropy did not suggest a clearly poorest model. Moreover, the VLMR-LRT suggested that five classes did not represent a meaningful improvement in fit over four classes (*p *= 0.119). We regarded that a five-class solution would not be interpretable due to sparse cell counts in the transition tables, leading to transition probability matrices that would not be estimable using maximum likelihood, and thus preferred a four-class solution. Based on mean values in each class (Table 2), we described the resultant four-class solution as comprising victims of bullying (class 1), perpetrators of aggression (class 2), extreme perpetrators of aggression (class 3) and neither victim nor perpetrator (class 4). Latent classes are also described in Fig. 1. Of note is that mental well-being was comparably poorer in all classes as compared to class 4 (neither victim nor perpetrator), suggesting co-occurring and similar impacts on mental well-being of both perpetration of aggression and bullying victimisation. Upon comparing four-class solutions at each of the follow-up measurement waves, we regarded that latent classes had metric similarity over time, though a metric invariance test was significant (*χ*^2^ = 8909.73, *df *= 24, *p *< 0.0001).

**Table 1:** Class selection

Classes	BIC	Entropy (%)	VLMR-LRT (*p* value)
1	105,864		
2	85,449	95.6	0.014
3	83,531	94.0	0.044
4	82,041	90.2	0.019
5	81,063	90.3	0.119
6	97,245	90.1	0.076

*BIC* Bayesian information criterion, *VLMR-LRT* Vuong-Lo-Mendell-Rubin likelihood ratio test

**Table 2:** Estimated latent classes from a four-class solution

Wave	Scale	Class 1: victim	Class 2: perpetrator	Class 3: extreme perpetrator	Class 4: neither	Variance
Baseline	GBS	6.99	2.64	3.59	1.16	2.72
	ESYTC	2.58	11.15	25.25	1.33	4.94
	SWEMWBS	20.95	21.38	19.41	25.10	32.01
24 months	GBS	6.39	2.03	3.70	0.90	2.09
	ESYTC	3.76	13.60	32.11	2.25	8.35
	SWEMWBS	20.42	21.47	19.39	24.44	32.60
36 months	GBS	6.11	1.84	3.49	0.69	1.83
	ESYTC	3.99	12.74	30.89	2.29	8.12
	SWEMWBS	19.39	20.74	20.32	23.82	34.81
Overall	GBS	6.49	2.01	3.07	0.99	1.95
	ESYTC	2.67	12.74	31.20	2.07	5.92
	SWEMWBS	21.72	22.09	20.23	24.11	27.74

*GBS* Gatehouse Bullying Scale, *ESYTC* Edinburgh Study of Youth Transitions and Crime school misbehaviour subscale, *SWEMWBS* Short Warwick-Edinburgh Mental Wellbeing Scale

In our latent transition model, intervention status was a significant overall predictor of transitions between classes (*χ*^2^ = 110.10, *df *= 24, *p *< 0.0001). Baseline distribution of classes was roughly similar between intervention and control group students. Intervention group students had a 12.9% probability of being victims (class 1), 6.8% probability of being aggression perpetrators (class 2), 1.2% probability of being extreme perpetrators (class 3) and a 79.1% probability of being neither victim nor perpetrator (class 4). Control group students had a 14.7% probability of being victims, a 7.7% probability of being aggression perpetrators, a 1.1% probability of being extreme perpetrators and a 76.4% probability of being neither victims nor perpetrators.

Transition matrices (Table 3) suggested that a key impact of the intervention was in the transitions of students who were extreme perpetrators from baseline to 24 months. In the control group, extreme perpetrators were equally likely to stay in this class (27.0% probability) or move to being aggression perpetrators (class 2; 25.0% probability) at 24 months. However, in the intervention group, the percentage of extreme perpetrators remaining in this class was much lower (5.4%), with a correspondingly larger increase in extreme perpetrators moving to being aggression perpetrators (class 2; 65.1%), i.e. a major decrease in aggressive behaviour and an improvement in mental well-being. Of interest is that there appeared to be a higher probability of extreme perpetrators moving to being neither victim nor perpetrator (class 4) in the control group (35.2%) as compared to in the intervention group (17.8%). Inspection of the transition matrices between 24 and 36 months suggested some later-breaking transitions from extreme perpetrators to aggression perpetrators in the control group (49.6% probability), which was greater than the comparable probability for the intervention group (37.0%). Between 24 and 36 months, more intervention students transitioned from being aggression perpetrators to neither victim nor perpetrator than in the control group (30.1% vs 22.3%). This is reflected in the marginal changes between timepoints in class distributions (see Table 4).

**Table 3:** Latent transitions

Control: baseline to 24 months						Intervention: baseline to 24 months					
		Destination class						Destination class			
		1: Victim	2: Perpetrator	3: Extreme perpetrator	4: Neither			1: Victim	2: Perpetrator	3: Extreme perpetrator	4: Neither
Sending class	1	0.410	0.115	0.001	0.474	Sending class	1	0.373	0.146	0.017	0.464
	2	0.047	0.598	0.068	0.287		2	0.026	0.595	0.114	0.266
	3	0.128	0.250	0.270	0.352		3	0.117	0.651	0.054	0.178
	4	0.044	0.089	0.016	0.851		4	0.036	0.074	0.015	0.875

Control: 24 to 36 months						Intervention: 24 to 36 months					
		Destination class						Destination class			
		1: Victim	2: Perpetrator	3: Extreme perpetrator	4: Neither			1: Victim	2: Perpetrator	3: Extreme perpetrator	4: Neither
Sending class	1	0.424	0.087	0.026	0.463	Sending class	1	0.449	0.059	0.022	0.470
	2	0.033	0.700	0.044	0.223		2	0.008	0.659	0.032	0.301
	3	0.035	0.496	0.081	0.388		3	0.027	0.370	0.200	0.403
	4	0.016	0.049	0.011	0.924		4	0.011	0.037	0.015	0.936

**Table 4:** Class distributions at each time

	Control				Intervention			
	Class 1: victim	Class 2: perpetrator	Class 3: extreme perpetrator	Class 4: neither	Class 1: victim	Class 2: perpetrator	Class 3: extreme perpetrator	Class 4: neither
Baseline	0.147	0.077	0.011	0.764	0.129	0.068	0.012	0.791
24 months	0.099	0.134	0.021	0.746	0.080	0.126	0.022	0.772
36 months	0.059	0.149	0.018	0.773	0.046	0.124	0.022	0.807

## Discussion

### Summary of Key Findings

Our analyses found evidence of intervention impacts in terms of a set of transitions from extreme perpetration of aggression to lower levels of perpetration. This was associated as well with improvements in mental well-being. These findings are interesting because they complement the main findings of the INCLUSIVE trial, which suggested no significant effects on perpetration of aggression at either follow-up while finding important effects on bullying victimisation at 36 but not 24 months. While our transition model does not contradict these findings of the original endpoint-based estimates of effect, it does add an important nuance to the picture: even if population-level perpetration of aggression is not impacted by this whole-school health intervention, such interventions may have important effects on the overall profile of risks and problems in those engaged in aggression, with concomitant impacts on mental well-being. Similar to the main findings of the INCLUSIVE trial, effects were small at the margins but would be expected to have substantial population impacts. Furthermore, our analyses indicate impacts of the intervention from baseline to 24 months that were not detectable in the primary analyses of differences in discrete end-points.

Our analysis contributed evidence to a developing understanding of how bullying victimisation and aggression perpetration co-occur. While many recent analyses in this area have focused on bullying victimisation alone (Ashrafi et al., 2020; Lee et al., 2020), fewer have also included measures of victimisation (Bettencourt et al., 2017; Ettekal & Ladd, 2019). We found that victimisation and perpetration separated clearly; that is, unlike previous analyses (Bettencourt et al., 2017; Ettekal & Ladd, 2019; Jenson et al., 2013), there was no co-occurring perpetrator/victim class, and that different levels of perpetration could be clearly distinguished, though our focus was on perpetration of aggression more broadly than on bullying perpetration alone. Unlike Ettekal & Ladd (2019), we did not separate aggression by type, and unlike Bettencourt et al. (2017), we did not include teacher report alongside peer report. It is possible, if not likely, that different observers may have systematically different patterns of perception; indeed, Bettencourt et al. (2017) found that teachers identified a joint aggressor-victim profile whereas children differentiated between these groups. Another plausible explanation for this finding is that our measure of aggression was more general in nature, extending beyond bullying perpetration as such.

Clinical standards do not exist to evaluate meaningful differences on measures of victimisation and perpetration used in this analysis, but patterns of conditional means suggest that the full range of each measure was represented across classes. However, estimates of clinically important change from the SWEMWBS support that differences of between 1 and 3 points are considered relevant and important (Shah et al., 2018). By this metric, it is clear that there are important differences in mental well-being between classes, with differences especially notable between extreme perpetrators and those neither perpetrating nor experiencing victimisation.

### Limitations

To retain a clear focus on the clustering of violent behaviours and states of mental health associated with these, our analyses focused on only three of the outcomes from the trial and did not examine latent classes or transitions concerning outcomes such as tobacco, alcohol and other drug use; sexual risk behaviour; psychological functioning; or health-related quality of life. These will be examined in subsequent papers. As is customary for LTAs, the analyses presented here were exploratory and not specified in the trial protocol; hence, type 1 error is possible. Our findings cannot automatically be generalised to other whole-school interventions in other schools or school systems. In addition, we were unable to establish metric invariance between measurement waves. While we regarded that indicator means were similar for each class between waves, a non-significant test of invariance would have provided more confidence of stability of measurement. This finding may, of course, also suggest as well that classes of bullying victimisation and aggression are not stable over a period of rapid developmental change. Finally, our analytic strategy accounted for the movement of students in and out of the study schools by including all measurements contributed. However, this meant that our analysis made the possibly strong assumption of data being missing at random and ‘explainable’ by other variables in our model. Assessment of missing data would need to disentangle which students missed a measurement wave because they were not present in school, because they refused to take part or because they were not enrolled in the school, which was not possible to account for in this analysis.

### Implications for Research and Policy

LTA provides a powerful method to better understand the effects of interventions, not only on different aspects of bullying but also more diverse risks and problems where these problems cluster within individuals. This is important for several reasons. First, LTA is particularly useful for understanding interventions like INCLUSIVE, which primary trial analyses indicated had emergent effects on a range of outcomes. The results of this LTA suggest that although existing conventional analyses of the effects of whole-school interventions point to the emergence of effects over time (Bonell et al., 2018; Patton et al., 2006), early effects may be detectable via analyses that examine multiple concurrent risks and problems. Second, LTA can inform which groups are most likely to benefit from population-level interventions. That is, even though INCLUSIVE had population-wide effects, these effects are driven in part by risk redistribution in transitions from highest-risk classes to lower-risk classes. By corollary, this has important implications for equity, in that latent transition models could signal either that interventions are impacting most strongly on the highest-risk groups, as was the case here, or possibly worsening or leaving behind such high-risk populations. Third, LTA provides a useful inductive tool to better understand how interventions work. This is a characteristic of the analysis process as a whole, in that (a) outcomes clustered into identifiable classes, (b) these classes were preserved over time and (c) the intervention led to changes in the probabilities of transitions over time. Identification of class metric stability and the transitions between classes thus provide an opportunity to infer mechanisms relating to outcome interaction and multilevel change processes, a point highlighted in our recent process evaluation (Warren et al., 2020). Future research should consider latent transitions through an equity lens, that is, with a view to understanding how transitions as a marker of intervention effectiveness might be different based on equity-relevant characteristics such as gender and socio-economic position.

Our findings suggest that this universal, whole-school intervention worked by redistributing individuals from higher to lower overall levels of risk across a range of inter-related outcomes. It is plausible that the restorative practices delivered as part of this intervention were successful in resolving conflicts and thereby reducing aggression and therefore improving the mental health of victims and perpetrators. That the intervention was effective in reducing clustered risks is also significant. Co-occurring risks interact within classes to produce adverse outcomes; for example, extreme aggression and poor mental well-being interact to produce negative long-term sequelae, such as criminal justice system involvement (Jennings et al., 2019). Thus, interventions that ‘shift’ co-occurring patterns of risk can have important long-term benefits over and above interventions that impact one risk pattern alone. However, our findings also suggested a potential group for which INCLUSIVE may have some risk of harm; specifically, between baseline and 24 months, control group participants in class 3 (aggression perpetrator) were more likely to move to class 4 (neither victim nor perpetrator) than intervention group participants in class 3. One potential mechanism for this is that structural school-level change may have entrenched the anti-social identities for some perpetrators, a ‘mirror image’ of the healthy context paradox described for victims of bullying (Garandeau & Salmivalli, 2019). Future interventions focusing on restorative practice could meet this challenge by enhancing supportive contexts for change, i.e. preventing this entrenchment by norming growth and development of character alongside denorming relational aggression.

Our results add to previous evidence from this trial on the implementation and outcomes of the INCLUSIVE intervention. The intervention was feasible and acceptable to deliver and as well as promoting multiple health benefits also increased student engagement in learning, and reduced student truancy and overall involvement in school discipline systems (Bonell et al., 2020). Our study adds to the evidence that whole-school interventions may be a pragmatic means of promoting students’ overall health in busy schools, in comparison to delivering multiple, separate classroom health curricula for each health topic (Bonell et al., 2014).
